# Optimizing O red blood cell concentrate usage in the emergency department in the era of patient blood management

**DOI:** 10.1016/j.htct.2024.05.008

**Published:** 2024-08-21

**Authors:** Louisiane Courcelles, Marie Pouplard, Orla Braun, Corentin Streel, Véronique Deneys

**Affiliations:** a Blood Transfusion Service, Cliniques Universitaires Saint-Luc, Avenue Hippocrate 10, 1200 Woluwe Saint-Lambert, 1200 Brussels, Belgium; b Emergency Department, Cliniques Universitaires Saint-Luc, Avenue Hippocrate 10, 1200 Woluwe Saint-Lambert, 1200 Brussels, Belgium

**Keywords:** Uncrossmatched transfusion, O-negative red blood cells, Emergency department, Patient blood management

## Abstract

**Background:**

Emergency transfusion may require the availability of O-negative red blood cell concentrates without pre-transfusion testing. At the Cliniques Universitaires Saint-Luc, the emergency department was used to having access to two decentralized O-negative red blood cell concentrates. This study aims to analyze the consumption of O-negative red blood cell concentrates in emergency situations both before and after the implementation of a novel strategy aiming at optimizing stocks. This strategy provides a combined allocation of one unit of O-positive red blood cell concentrate and one unit of O-negative red blood cell concentrate decentralized in the emergency department and reserve the transfusion of the negative unit only to under 45-year-old women and under 20-year-old men.

**Materials and Methods:**

A retrospective study was conducted of the transfusion and medical records of all patients who received immediate transfusions in the emergency department without pre-transfusion testing between 2008 and 2022.

**Results:**

A total of 193 patients received O red blood cell concentrates without pre-transfusion testing in emergency situations between 2008 and 2022. During the first 24 h of hospitalization, 354 O-negative units were transfused. Mean ratios of number of O-negative bags between 2008 and 2020 was 1.98 unit/patient. After implementation of the new strategy, the ratio in 2021 was 1.46 unit/patient and drastically decreased in 2022 to 0.79 unit/patient.

**Conclusion:**

In situations of emergency, allocating O-negative units only for women younger than 45 years and men younger than 20 years could have saved 85% of O-negative red blood cell concentrates transfused (303/354) yet balancing the immunological risk. Limiting the number of delocalized units of O-negative red blood cell concentrates in the emergency department seems to lower O-negative consumption. With this strategy, the units spared could have been transfused to patients with greater needs (e.g., sickle cell patients or chronically transfused patients).

## Introduction

Immediate transfusion of red blood cell concentrates (RBCs) is a lifesaving measure in the emergency department (ED). At the Cliniques Universitaires Saint-Luc (Brussels, Belgium), a tertiary hospital, the ED is recognized as a trauma center. According to guidelines of the Association for the Advancement of Blood & Biotherapies (AABB),^1^ Blood Transfusion Services (BTS) must have a procedure in place for the urgent release of RBCs before the completion of compatibility testing. Immediate transfusion involves the use of uncrossmatched RBCs, typically O-negative RBCs which are known as the ‘universal donor’. To facilitate this process, two units of O-negative RBCs were kept in a monitored refrigerator in the ED.

In recent years, the shortage of group O-negative RBCs, exacerbated by the COVID-19 crisis which has had a dramatic effect on stocks, has become a significant clinical issue for the BTS.^2^ To address this problem, solutions had to be found to optimize the supply, working collaboratively between the blood collection establishment (BCE), the BTS and clinical departments consuming blood. Due to a major shortage of O-negative RBC units in 2021, the BTS decided to stock only one unit of O-negative RBCs with one unit of O-positive RBCs in the ED.

The objective of this study was to analyze past consumption of O RBCs in the ED and to compare this to the new strategy implemented to optimize stocks while balancing the immunological risk for the patient. It also considered the necessity of delocalized RBC products in an emergency department.

## Materials and methods

A retrospective review was conducted of all patients (n = 193) who received uncrossmatched packed O red blood cells (pRBCs) between January 1, 2008 and December 31, 2022 at the ED of Cliniques Universitaires Saint-Luc in Brussels, Belgium. Data collected included patient blood group, antibody screening results, number and blood group of subsequent pRBC transfusions, age, gender and length of hospitalization. Transfusion data of subsequent antibody appearance was also evaluated in patients who had at least one antibody screening performed after the transfusion during the 15-year study period.

The indicator used to evaluate blood consumption was referred to as the ‘ratio’ which assessed the number of urgently RBC units per transfused patient during a given period.

Descriptive statistical analysis was performed with Microsoft® Excel 2016. The distribution of blood groups in the present cohort was compared with those obtained in a larger Caucasian population,^3^ the significance of differences observed were individually assessed using a Pearson Chi-square association test. The p-value reflects the probability of both populations being similar (significant at 0.05).

This study was approved by the ethics committee of the Cliniques Universitaires Saint-Luc (ref. 2022/30NOV/458).

## Results

### Demography

**Figure 1:**
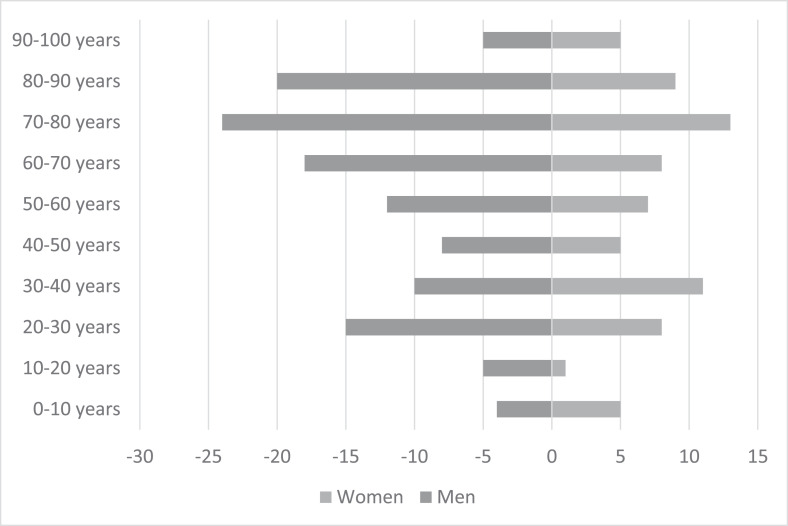
Population pyramid of patients transfused with O-negative RBC units at the emergency department between 2008 and 2022 (n = 193).

Between 2008 and 2022, 193 patients were transfused with uncrossmatched O pRBC units at the ED (121 - 63% male; 72 - 37% female) (Figure 1 and Table 1). The mean age of patients was 56 years with 14% of women under 45 and 5% of men under 20 years. The survival rate was 61% during the first 24 hours of admission at the ED and 48% after 15 days of hospitalization.

**Table 1:** Demographic characteristics of patients admitted and transfused with uncrossmatched units in the emergency department between 2008 and 2022 (n = 193).

Admission feature	
Mean Age (min-max)	56 (8 months-98 years)
Male - n (%)	121 (63%)
Women of <45 years - n (%)	27 (14%)
Males of < 20 years - n (%)	9 (5%)


Survival	
Day 1 - n (%)	117 (61%)
Day 15 - n (%)	92 (48%)

### Blood group

The distribution of ABO blood groups (summarized in Table 2) was similar to that observed in the Caucasian population.^3^ Special attention was paid to Rh -2-3 (Rh ccee) patients: 10% of emergency transfused patients were Rh group rr (ccddee) and 10% of patients were R_0_r (ccDee), these two populations were statistically different from those observed in our reference Caucasian population. Prior to admission, 20% already had had their blood group identified and the blood groups of 7% were not identified due to their early death.

**Table 2:** Blood group distribution among the patients of this study compared to a reference Caucasian population.

Blood group	Frequency in our study's patients (n = 193)	Frequency in a reference Caucasian population ^(3)^	p-value
O	41.9%	44.7%	0.635
A	37.8%	43.5%	0.11
B	10.3%	9%	0.529
AB	3.6%	3.6%	0.962
ABO *rr* (ccddee)	10%	15.8 %	0.025
ABO *R_0_r* (ccDee)	10%	2.1%	<0.0001
At risk of immunization if transfused with O-negative	23.5%		
*R_1_R_1_* (CCDee)	22%	16,2%	0.039
*R_2_R_2_* (ccDEE)	1%	2.3%	0.238
*R_1_R_z_* (CCDEe)	0.5%	0.06%	0.013
Blood already determined at admission (other than O rr or *R_0_r)*	20%		
Blood group Never determined (due to death)	7%		

### Blood unit distribution

**Figure 2:**
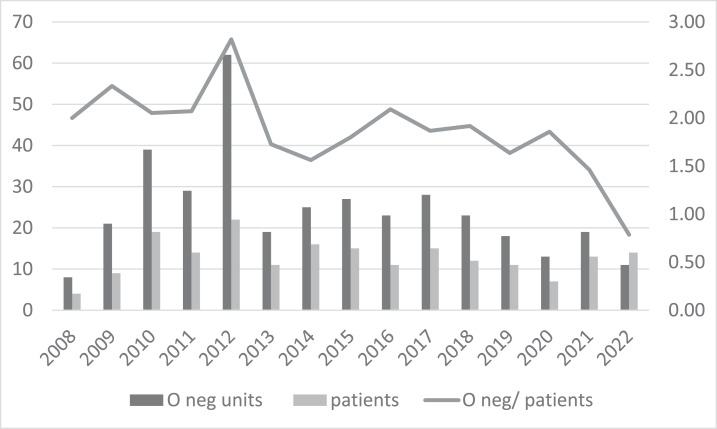
Evolution of distribution of O-negative units transfused to 193 patients in the emergency department between 2008 and 2022.

In February 2021, the BTS decided to store only one O Rh-negative pRBC and one O Rh-positive pRBC unit due to O negative shortages in Belgium. No uncrossmatched O-positive unit was given in the ED in 2021 however, of the 14 patients transfused in 2022, 6 patients received one uncrossmatched O-positive unit each and 3 patients received both the O-negative and O-positive units that were stored in the ED. Mean ratio of the number of O Rh-negative bags between 2008 and 2020 was 1.98 unit/patient. The ratio in 2021 was 1.46 unit/patient; this drastically decreased in 2022 to 0.79 (see Figure 2).

The consumptions of pRBCs during the first 24 hours (Day 0) and after (Days 1-30) were evaluated (Table 3*).* 62 patients (32%) received only one or two units of O RBCs (immediately available in the ED) during the first 24 hours. Among them, 32 patients died within the first 24 hours but 17 patients (9%) received only these pRBCs during all their hospitalization. 35 patients (20%) received massive transfusions of more than ten pRBC units within 24 hours according to the International Society of Blood Transfusion (ISBT) definition^4^ with only one being massively transfused with O-negative pRBC unit.

**Table 3:** Number of patients transfused with 1, 2 or more pRBC units during the first 24 hours and during all the hospitalization.

Number of pRBC units transfused	Number of patients (n = 193)	No. of survivors after 24 hours – n (%)
1 or 2 pRBC units within the first 24 hours	62	32 (52)
2 to 10 pRBC units within the first 24 hours	96	67 (70)
>10 pRBC units within the first 24 hours	35	19 (54)
1 or 2 pRBC units during all the hospitalization (>24 hours)	**17**	

pRBC: packed red blood cell

### Indirect antiglobulin test and serological follow-up

Of the 193 patients admitted at the ED, 4 patients had positive indirect antiglobulin tests (IATs) at admission; Patient 1 had anti-E antibodies, Patient 2 had anti-E and anti-Cw antibodies, Patient 3 had anti-E, anti-c and anti-Kpa antibodies and Patient 4 had anti-e antibodies. Patient 3 was already known before the hemorrhagic episode. Patient 4 received four O-negative units then switched to O-positive RBCs (5 units) but died within the first 24 hours.

Of all the patients, 133 (69%) did not have a follow-up IAT performed at our institution during the 12-year study period after the transfusion. We do not have information concerning their subsequent immunological status. Conversely, 60 patients had at least one repeated antibody screening with a median of 17 days after the episode (interquartile range: 7-183 days). Of all the patients who were tested, only one patient developed an allo-antibody (anti-Cw - Table 4)*.*

**Table 4:** Serological Follow-up data (n = 193).

Immunological status	
Positive IAT at admission	2.1%
Patient IAT Screen after transfusion episode	31%
Delay between transfusion episode and IAT	Median: 17 days Interquartile range: 7-183 days
Positive IAT after transfusion episode	1.6% (1 patient in 60 patients)

IAT: Indirect Antiglobulin Test

## Discussion

Between 2013 and 2021, after the introduction of PBM in Belgian hospitals, the number of blood units transfused decreased by 13.5%.^5^ However, this decrease was mainly due to the extra A and AB RBC units, while, on the contrary, the proportion of O RBC units increased slightly by 5.2%, thus causing an imbalance between the needs and resources.^5^^,^^6^ This phenomenon was also observed in other countries.^5^^,^^7^ The overconsumption of these O-negative RBC units leads to a shortage that is difficult to manage for BTSs.

In our tertiary care Belgian hospital, the relative distance between the BTS and the ED led the hospital to safely store two O Rh-negative pRBC units in the ED in case of an acute need of blood. Due to the major shortage that hit Belgium in the autumn of 2021, it was decided to evaluate the consumption of O RBCs.

In February 2021, we proposed a new strategy for allocating uncrossmatched O units for acute need of transfusion in the ED based on the age, gender and medical records of patients.^6^ Importantly, there were no changes in the clinical protocols or admission profiles of patients compared to the previous strategy. The first characteristics to be considered were admission features: gender and age. Our cohort showed that the majority of urgent transfusions were for men (63%) and the mean age of all patients was 56 years: 201 O Rh-negative units were given to over 20-year-old males. However, the patient population most at risk for the deleterious effect of anti-D alloimmunization are females of childbearing age. The threshold of <45 years was approved by the transfusion committee of the Cliniques Universitaires Saint-Luc. Of the 365 O-negative units transfused at the ED, 74 units were given to women over 45 years old (Table 5) and so 74 units could have been saved if these patients had been transfused directly with O Rh positive uncrossmatched units.

**Table 5:** Simulation of the new strategy applied on the cohort (n = 193) with the ‘non-indication’ of uncrossmatched transfusion of O-negative unit.

Non-indications for uncrossmatched O-negative RBC transfusion	O-negative bags consumed
Women >45 years	74 (20%)
Men >20 years	201 (55%)
Already known - ABO RH group other than O Rh-negative and *R_0_r* (checked in the medical record)	28 (8%)
Total	303 (85%)

Anti-D alloimmunization in women of childbearing potential may cause hemolytic disease of the newborn in cases of a future pregnancy with an Rh-positive fetus. However, this risk should also be put into perspective. The overall fetal death rate in an RhD-childbearing woman who has been transfused with RhD-negative RBC units, survived her trauma, been alloimmunized, and then carries an RhD-positive fetus has been calculated to be 0.3%, mainly due to the excellent diagnostic and therapeutic modalities available at referral centers.^8^ If a woman of childbearing age receives RhD-positive RBCs outside of pregnancy, administration of anti-D immunoglobulin should be considered to reduce the risk of anti-D alloimmunization.^9^

In case of an Rh incompatible transfusion in a patient with anti-D antibodies, a transfusion reaction of the extravascular hemolysis type is to be feared. Most Rh antibodies are IgG antibodies that do not activate complement. Instead, the donor's RBCs will be eliminated by the reticuloendothelial system (mainly macrophages in the spleen and liver). This transfusion reaction is much less severe than the intravascular hemolysis encountered in ABO incompatible transfusions. In the former case, extravascular destruction of RBCs is slower and more controlled than intravascular hemolysis, and very little quantity of free hemoglobin is released into the circulation or excreted in the urine. The liver can keep up with the increased production of bilirubin and jaundice rarely occurs. Therefore, the main symptoms of this type of reaction are fever and chills.

Nevertheless, it should be noted that the administration of O-negative RBCs is not without risks for certain patients: 23.5% of patients are at risk of alloimmunisation due to a Rh incompatibility (Table 2). Although in our study, only one patient developed an allo-antibody (anti-Cw), we most certainly underestimated this risk because of the lack of systematic antibody screening after the transfusion episode (only 31% of patients). In case of transfusion without pretransfusion laboratory testing, systematic antibody screening should be assessed between seven days and two weeks after the transfusion episode to detect any presence of anamnestic response or alloimmunisation.^10^

The myth of O-negative as the erythrocyte component of choice for an unknown blood type is currently being challenged. Transfusing O-negative is not a universal solution and comes with the risk of immunization: some D-positive groups are at risk of developing anti-c and anti-e antibodies after being transfused with O-negative blood. On the other hand, there is a general fear that by administering Rh-positive RBCs, the patient will become alloimmunised with an anti-D antibody. These notions must be put into perspective: the rates of alloimmunization reported in the literature for Rh-negative ‘trauma’ patients who have been transfused with Rh-positive blood vary greatly, from 7.8%^11^ in the most recent study to as much as 50%.^12^, ^13^, ^14^, ^15^ To put these figures in perspective, the Recipient Epidemiology and Donor Evaluation Study-III (REDS-III) of registry analysis showed a rate of alloimmunization after transfusion of 6.67% for all transfused patients combined.^16^ In the general population, the rate of positive antibody screening is 2.06% (subjects were not necessarily transfused). In general, certain clinical conditions are associated with a higher risk of alloimmunization, including sickle cell disease^17^ and myelodysplastic syndrome.^18^ Conversely, other disorders are associated with a lower rate of immunization such as leukemia.^19^ The same study showed that elderly subjects, Rh-negative patients, and female subjects are at greater risk of having a positive IAT. In patients classified as ‘trauma’, there seems to be an inverse correlation between the number of units transfused and the risk of immunization; patients who developed anti-D appeared to have been less severely injured.^11^^,^^20^

In this study, we demonstrated that the policy of having two O-negative RBC units in a monitored refrigerator in the ED led to unnecessary consumption of O-negative blood because of the easy local accessibility. This assumption was based on the high number of transfusions of only one RBC unit of strictly O-negative RBCs (9% of all patients) during all the hospitalization and the fact that 20% of all patients had an already known group different than O Rh-negative or O *R_0_r*. Even though it would be ideal to abandon the storage of RBCs in the ED, in 2021 we decided to replace one of the two RBC units available with O-positive blood for immediate transfusion because of the O-negative blood shortage. In case of unknown blood group and acute need of transfusion (that cannot wait for blood group determination), we allowed the administration of a maximum of two units of RBCs in the ED to over 45-year-old female and over 20-year-old male patients to have time to obtain the patient's blood group and transfuse into that group (according to the guidelines of the Australian & New Zealand Society of Blood Transfusion - ANZSBT).^21^ Since the change in practice, the consumption of O-negative bags has more than halved (the ratio of O-negative pRBC units/patient was 1.98 between 2008 and 2020, 1.46 in 2021 and 0.79 in 2022). The observed effect cannot be attributed to clinical protocols or admission standards, as there were no significant modifications in hospital policies preceding or following the implementation of this strategy. Among the subset of massively transfused patients who might influence the ratio, only one out of 193 individuals received more than ten units of O-negative pRBCs before the change compared to none in the post-strategy cohort. This finding supports the conclusion that the reduction in O-negative blood consumption can be attributed to the strategic intervention.

This study presents a number of limitations due to its retrospective design. A significant number of patients did not have serological follow-ups after the transfusion episodes and this situation may underestimate the number of immunized patients. Medical and transfusion data were restricted to those obtained in our hospital, Belgium does not have a shared transfusion record yet. Finally, the period of the study after the implementation of the change of practice was relatively short.

## Conclusions

The use of O-negative pRBCs in EDs should be optimized in order to reserve blood for patients who need it the most, especially in the case of massive transfusions and during O-negative blood shortages. We observed that the availability of O RBC units directly in the ED seems to have a negative impact on O-negative consumption.

Clear protocols describing the situations in which patients can be transfused with O-positive blood should be implemented to enable the right product to be given to the right patient and promote PBM.

## Conflicts of interest

The authors affirm that they have no conflicts of interest to declare.
